# National prevalence and risk factors for tungiasis in Kenya

**DOI:** 10.1186/s40249-023-01131-x

**Published:** 2023-09-18

**Authors:** Lynne Elson, Christopher Kamau, Sammy Koech, Christopher Muthama, George Gachomba, Erastus Sinoti, Elwyn Chondo, Eliud Mburu, Miriam Wakio, Jimmy Lore, Marta Maia, Ifedayo Adetifa, Benedict Orindi, Phillip Bejon, Ulrike Fillinger

**Affiliations:** 1 Kenya Medical Research Institute-Wellcome Trust Research Programme, Kilifi, Kenya; 2 Nuffield Department of Medicine, University of Oxford, Oxford, UK; 3 Department of Health, Muranga, Kenya; 4 Department of Health, Kericho, Kenya; 5 Department of Health, Makueni, Kenya; 6 Department of Health, Nakuru, Kenya; 7 Department of Health, Samburu, Kenya; 8 Department of Health, Kilifi, Kenya; 9 Department of Health, Kajiado, Kenya; 10 Department of Health, Taita Taveta, Kenya; 11 Department of Health, Turkana, Kenya; 12 London School of Hygiene and Tropical Medicine, Keppel Street, London, UK; 13 International Centre for Insect Physiology and Ecology (Icipe), Nairobi, Kenya

**Keywords:** Tungiasis, Neglected tropical diseases, Prevalence, Disease burden, Kenya

## Abstract

**Background:**

Tungiasis is a highly neglected tropical skin disease caused by the sand flea, *Tunga penetrans*, the female of which burrows into the skin, causing pain and itching. The disease occurs throughout South America and sub-Saharan Africa but there are few systematic data on national disease burdens. The tungiasis research community is keen to develop survey methods to fill this gap. Here we used a school-based, thorough examination method to determine the prevalence and risk factors for tungiasis in Kenya.

**Methods:**

We conducted the first nationally representative survey of tungiasis, including nine counties covering the major ecological zones of Kenya. A stratified multistage random sampling was used to select 22 primary schools from each of the nine counties and to select up to 114 pupils aged 8 to 14 years in each school. Pupils were examined thoroughly for tungiasis. Two surveys were conducted, the first between May and July 2021 and the second between October 2021 and April 2023 when pupils were also interviewed for risk factors. Mixed effect logistic regression models were used to test associations of independent variables with tungiasis using the school as a random effect.

**Results:**

The overall prevalence of tungiasis in the first survey was 1.35% [95% confidence interval (*CI)*: 1.15–1.59%], and 0.89% in the second survey. The prevalence ranged from 0.08% (95% *CI*: 0.01–0.59%) in Taita Taveta county to 3.24% (95% *CI*: 2.35–4.44%) in Kajiado county. Tungiasis infection was associated with county of residence, male sex [adjusted odds ratio (a*OR*) = 2.01, 95% *CI*: 1.52–2.67], and lower age (*aOR* = 0.81, 95% *CI*: 0.75–0.88). For the first time we demonstrate an association with attending public schools rather than private schools (*aOR* = 5.62, 95% *CI:* 1.20–26.22) and lower socioeconomic status (*aOR* = 0.10, 95% *CI*: 0.03–0.33). Using a rapid screening method of the top of feet only, would have missed 62.9% of all cases, 78.9% of mild cases and 20.0% of severe cases.

**Conclusions:**

Tungiasis is widely but heterogeneously distributed across Kenya. School-based surveys offer an efficient strategy for mapping tungiasis distribution.

**Graphical Abstract:**

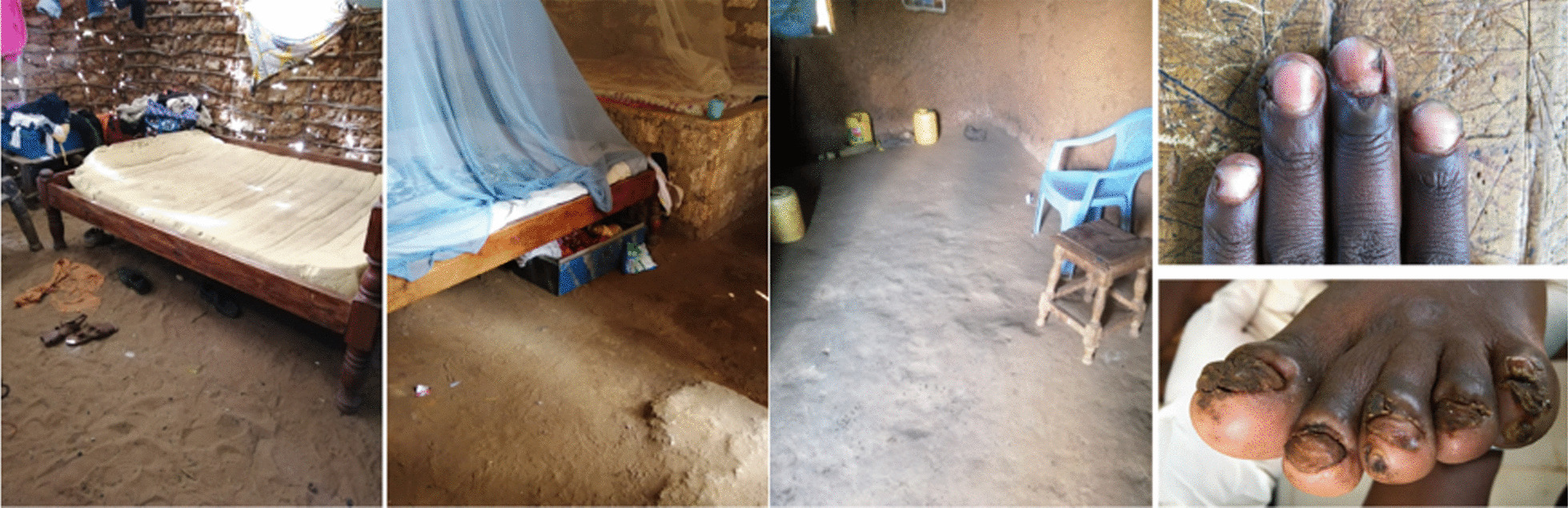

**Supplementary Information:**

The online version contains supplementary material available at 10.1186/s40249-023-01131-x.

## Background

Tungiasis is a skin disease caused by adult female sand fleas, which burrows into the skin, mostly of the feet. Once embedded, the female grows 2000-fold over 7 days as eggs develop in the abdomen causing inflammation, and pain and itching which in turn cause difficulty in walking, sleeping and concentrating.

The disease mostly affects children, the elderly and disabled people in resource-poor, marginalized populations in Central and South America and sub-Saharan Africa [1]. The main risk factors for tungiasis have been identified from multiple studies in resource-poor communities having a high prevalence of tungiasis. The factors are all poverty-related including living in a house with an unsealed earthen floor [2–6], ownership of pigs or dogs [2, 7–9], not wearing shoes [7, 9, 10] and not always washing feet with soap [3–5].

In 2020, tungiasis was added to the World Health Organization’s (WHO) Roadmap for Neglected Tropical Diseases (NTDs) under scabies and other “ectoparasitoses” and is targeted for control [11]. Individual surveys have demonstrated prevalence to range from 7% in a village in coastal Kenya [12] to 62.8% in Napak District in northeastern Uganda [13], but there are no systematic data on the overall disease burden and distribution in any one endemic country. This information is critical to plan the scale of and to target interventions to the right communities.

As countries prepare to assess their disease burden, discussions are being had as to how best to do this, using village or school-based surveys, online forms, or mobile apps to be used by health workers, community health volunteers or non-governmental organisations. Full examinations are laborious, and new protocols have been proposed using rapid observations. Unlike many other skin diseases, tungiasis is quite distinctive in appearance making it relatively simple to diagnose and unlikely to be confused with another skin disease. One option which has been proposed in the literature is a rapid screening technique, lining school pupils up and quickly observing the top of their feet for periungual lesions [14]. This method has not been subjected to a rigorous comparison with full examinations in a large population with a dark skin.

In this study, we aimed to determine the national prevalence of tungiasis in Kenya, to assess whether a rapid survey method would be sufficient for future surveys, assess disease severity and to determine explanatory factors for infection to identify possible interventions that could reduce the disease prevalence.

## Methods

### Study design

This study consisted of two cross-sectional surveys of primary school pupils with the first survey conducted between April and June 2021 which is the main wet season in most counties. The second survey was conducted in a different group of randomly selected schools from September 2021 to April 2023 but varying in duration from county to county and spanning dry and wet seasons (Table 2). The surveys focused on pupils aged 8 to 14 years since this is the age group previously established to be most affected [2, 12] and able to answer questions about their home and family.

### Study implementation

To build capacity for tungiasis surveillance, the surveys were conducted by partnering in each county with the Departments of Health and Education. In Kenya management of health services is devolved to the 47 counties which is managed through a County Health Management Team (CHMT) headed by a county executive, a director and chief officer. The Department of Health nominated a CHMT member, usually the County Disease Surveillance Officer or NTD Coordinator, to coordinate the study and recruit a team of five people to carry out the surveys. The principal investigator reviewed curricula vitae of potential team members who were mostly public health graduates. The principal investigator trained all team members and the coordinator. In every school the public health officer responsible for the area joined the team and two community health volunteers were trained to assist. The Department of Education gave approval for the study and provided school lists for each sub-county.

### Sample size

The sample size for assessing tungiasis prevalence in Kenya was calculated using a simple random sample formula [15] taking into account the design effect due to clustering [16], the stratified sampling approach, and available resources. The main outcome was tungiasis prevalence in Kenya. Previous studies have reported prevalence of tungiasis to range from 15–60% within schools, thus we assumed an average prevalence of 30%. For a study with 95% confidence levels (Z_α/2_ = 1.96), 95% precision, a cluster size of 110 and assuming an intraclass correlation coefficient (ICC) of 0.125 (based on calculations for soil-transmitted helminths), we estimated a sample size of 11,010 pupils spread across the 5 ecological zones (2 counties in each). If we maintained a cluster size of 110, cluster being one primary school, we would need 20 schools in each climate zone. Since one county declined to participate the sample size of 11,010 was then re-distributed across 9 counties requiring 11 schools per county and 114 pupils were targeted in each school.

### Study population

**Fig. 1:**
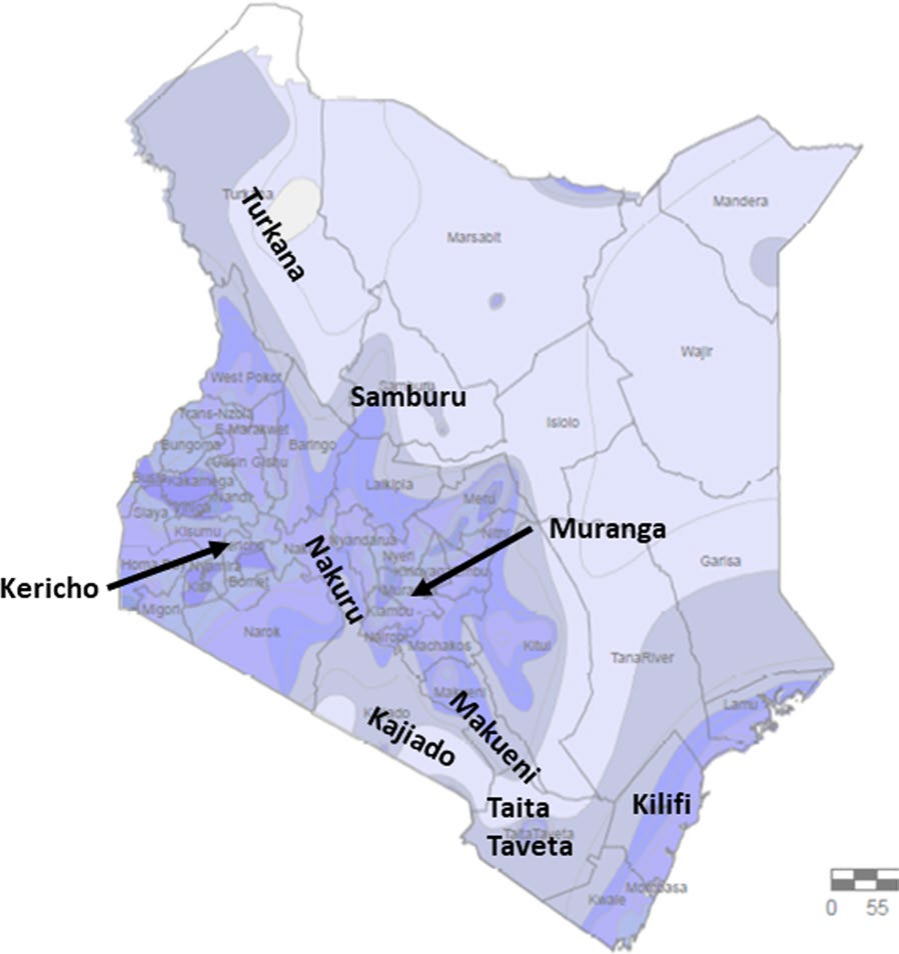
Rainfall map of Kenya and counties enrolled in the study (bold type). Map generated by C. Nyundo, KEMRI-Wellcome Trust Research Programme in ArcGIS 10.8.2 using rainfall data from Kenya Meteorological department website https://meteo.go.ke/

Nine counties of the 47 in Kenya were purposively selected to represent the main climatic zones. These included: Kericho and Muranga as humid; Nakuru as semi-humid; Kajiado, Kilifi and Makueni as semi-arid; Taita-Taveta and Samburu as being mostly arid; and Turkana as very arid (Fig. 1).

Stratified random sampling was used to select 22 primary schools in each participating county ensuring coverage of all sub-counties, using lists provided by the County Department of Education. Within each selected school an equal number of boys and girls were quasi-randomly selected by asking the pupils to assemble in three age groups; 8 and 9 years; 10 and 11 years; 12 to 14 years, and by sex within each age group. For each of these age/sex groups, every n^th^ pupil (*n* = total number in the group/19) was selected until 19 was selected, giving a total of 114 overall.

### Clinical assessment procedures

The feet of the 114 children in each participating school were washed and dried and systematically examined by trained field workers for the presence of tungiasis as described previously [17]. Those pupils found to have fleas in their feet, were assessed for intensity of infection by counting the number of embedded fleas (live, dead, manipulated lesions and flea clusters). For infected pupils, field officers also recorded whether the infection could be detected by simply looking at the top of the feet (peri-ungual areas) alone.

Infected pupils were also examined for associated morbidity recording the presence of symptoms that are easily identified by non-clinical field officers; desquamation, fissures, ulcers and abscess for acute symptoms and hyperkeratosis, deformed nails and lost nails for chronic symptoms. Symptoms normally recorded by previous studies [18] but omitted here were oedema, erythema, warmness and peri-ungual hyperkeratosis. All pupils were also observed for any obvious disability and signs of other skin diseases or abnormalities.

### Explanatory variables

All schools were categorized either as public or private during the survey and further as urban or rural based on housing density in their location. In the second survey, six pupils without infection were randomly selected in each school using the paper lottery method and were invited for interview together with all infected pupils. An opt-out informed consent process was used as described further in the Ethics section below. All selected pupils were interviewed using a structured questionnaire collecting information on demographic, behavioural, social, parental, and economic factors.

### Data analysis

All analyses were conducted in Stata IC version 15.1 (Stata Corp LLC, College Station, Texas, USA). Figure S1 in Additional materials (Additional file 1), illustrates the pupil selection and numbers included for each analysis. The prevalence of tungiasis (proportion of examined participants infected with tungiasis in their feet or hands) and 95% confidence intervals (*CI*s) were calculated for the country as a whole, for each county and for each school based on data collected from the first cross-sectional surveys only. A chi-squared test was used to test for a significant difference between counties. The second round of cross-sectional surveys in the counties was conducted over a period of one and a half years due to logistical challenges and therefore data could not be used to obtain a point prevalence.

For analysis of infection intensity and associated symptoms, a new dataset was created merging all infected participants from both surveys. The infection intensity for each infected individual was calculated as the sum of all flea stages (live, dead and manipulated) on both feet, plus the number of flea clusters multiplied by 5 (as an estimate for an average number of fleas/cluster). A total clinical score was calculated for each patient by summing the number of areas on the feet (each foot being divided in to 9 areas: 5 toes, medial side, lateral side, heel and sole) exhibiting acute symptoms (desquamation, fissures, ulcers and abscess) to a maximum score of 72, and chronic symptoms (hyperkeratosis, deformed nails and lost nails) to a maximum score of 38, to give an overall maximum clinical score of 110.

A scatter plot was created of infection intensity by clinical score for all infected cases and a polynomial regression line fitted. Patient disease severity was then classified using a recently developed threshold [17] of 10 embedded fleas, less than 11 fleas being considered mild disease and more than 10 fleas being severe disease. To determine the relationship between county prevalence and disease severity a scatterplot and fitted linear regression was created of the number of cases in each county from both surveys combined and the percent of those cases that were severe.

To test for associations between tungiasis infection status (infected and not infected) and possible explanatory variables, two-level mixed effects logistic models were used with an exchangeable correlation matrix and school ID number used as random effect. Sex of the pupil, school class, age at survey, disability status, having other skin disease(s), school type (public/private), school location (urban/rural), socio-economic status (SES) of the pupil, and county were included as fixed effects. County was not considered random because (1) their number was small, and (2) they were not selected at random. Initially, univariable analyses were run for each explanatory variable and then those with a *P*-value less than 0.2 were included in the multivariable model. Backward elimination, sequentially excluding variables was used to develop the final model using Akaike information criteria (AIC) to compare the models. Models with lower AIC values were preferred. Additional consideration on variable selection was expert knowledge on potential relationships with the outcome. Interactions terms were explored but were not significant and were therefore not included in the models. Wald tests were also run for validity of variables included in the final model and are presented as p-values in the footnote to the table. Univariable effects were presented as odds ratios (*OR*) and multivariable effects as adjusted odds ratios (*aOR*). The SES variable was derived through polychoric principal component analysis as described in Additional material S5 (Additional file 1).

The intra-cluster correlation coefficient (ICC) was calculated for the full data set with 21,467 pupils to guide future study designs and sample size calculations and to assess the need for a multilevel analysis. The ICC measures the relatedness of measurements from pupils within a school and ranges from 0 (pupils within a school are as heterogeneous as pupils between schools) to 1 (pupils within a school show identical measurements) [19].

## Results

During the first cross-sectional surveys between May and September 2021, 99 schools were surveyed with a total of 10,865 pupils examined for tungiasis (Table 1). In the survey between October 2021 and April 2023, 97 schools were surveyed with a total of 10,600 pupils examined.

**Table 1:** Characteristics of the study population

	Survey 1	Survey 2	Total
Months of survey	May–July 2021	Oct 2021–Apr 2023	
Number of counties	9	9	9
Number of schools	99	97	196
Public/private	94/5	85/12	179/17
Rural/urban	76/19	84/13	160/32
Number of pupils screened	10,865	10,600	21,467
Male sex,* n* (%)	5337 (49.1%)	5238 (49.4%)	10,576 (49.3%)
Age in years, mean (*SD*)	10.8 (1.96)	10.8 (1.94)	10.8 (1.95)
Number with disability (%)^a^	50 (0.5%)	51(0.5%)	101 (0.5%)
Number with other skin abnormality (%)^a^	425 (3.9%)	513 (4.8%)	938 (4.4%)

^a^not assessed by a clinician

### Prevalence of tungiasis

The overall prevalence of tungiasis during the first survey was 1.35% (95% *CI*: 1.15–1.59%, Table 2). Of the infected pupils, 7 (4.8%) had fleas only in the hands but not in their feet. The prevalence varied markedly between counties, from 0.08% (95% *CI*: 0.01–0.59%) in Taita Taveta to 3.24% (95% *CI:* 2.35–4.44%) in Kajiado. During the second survey only 0.89% of pupils were found infected (Table 2).

**Table 2:** Prevalence of tungiasis by county and survey

	Survey 1					Survey 2				
County	Survey months	Number of schools surveyed	Schools with at least 1 case, *n* (%)	Number of pupils examined	Pupil infection rate, % (95% *CI*)	Survey months	Number of schools surveyed	Schools with at least 1 case, *n* (%)	Number of pupils examined	Pupil infection rate, % (95% *CI*)
Turkana	May, Jun, July 2021	11	4 (36.4)	1189	0.67 (0.34–1.34)	Oct, Nov, Dec 2021, Jun 2022	11	2 (18.2)	1212	0.25 (0.08–0.76)
Samburu	May, Jun, July 2021	11	5 (45.5)	1220	1.72 (1.12–2.63)	Oct, Nov, Dec 2021, Jan, May 2022	11	3 (27.3)	1129	0.89 (0.48–1.64)
Kericho	May, Jun, July 2021	11	4 (36.4)	1222	0.65 (0.33–1.30)	Oct, Nov, Dec 2021,	11	4 (36.4)	1188	0.51 (0.23–1.12)
Muranga	May, Jun, July 2021	11	7 (63.6)	1240	2.02 (1.37–2.97)	Oct, Nov, Dec 2021, Feb 2022	11	9 (81.8)	1220	2.05 (1.39–3.02)
Nakuru	May, Jun, July 2021	11	5 (45.5)	1218	2.63 (1.86–3.69)	Oct, Nov, Dec 2021, May 2022	11	3 (27.3)	1222	0.65 (0.33–1.30)
Kajiado	May, Jun, July 2021	11	4 (36.4)	1143	3.24 (2.35–4.44)	Nov, Dec 2021, Jan, Feb, Sep 2022	11	4 (36.4)	1225	0.49 (0.22–1.09)
Makueni	May, Jun, July 2021	11	6 (54.5)	1229	0.65 (0.33–1.30)	Oct, Nov, Dec 2021, May 2022	11	2 (18.2)	1210	0.41 (0.17–0.99)
Taita Taveta	May, Jun, July 2021	11	1 (9.1)	1197	0.08 (0.01–0.59)	Oct, Nov, Dec 2021,	11	2 (18.2)	1215	0.16 (0.04–0.66)
Kilifi	Jun, Jul, Sep 2021	11	5 (45.5)	1208	0.58 (0.28–1.21)	May, Sep, Oct 2022, Mar, Apr 2023	9	6 (66.7)	979	3.06 (2.15–4.34)
TOTAL		99	41 (41.4)	10,866	1.35 (1.15–1.59)		97	35 (36.1)	10,600	0.89 (0.73–1.10)

*CI* Confidence interval

The prevalence also varied considerably across schools, with 41 of 99 schools (41.4%) in the first survey, and 35 of 97 schools (36.1%) in the second survey, having at least one case (Table 2). The majority of the affected schools (82.9%) having a prevalence of less than 5%, and only 3 schools having a prevalence of more than 10% (Table 2, seen in survey 1). There was heterogeneity within county: for instance, in Kajiado and Samburu counties most of the cases (67.6% and 68.4% of all cases in the county, respectively) were recorded from only one of eleven schools. On the other hand, in Muranga county, the disease was more evenly distributed with infected pupils identified in seven schools with only one school having more than five cases.

### Disease severity

To assess intensity of infection and morbidity, data on all infected pupils were combined for both surveys (*n* = 242). The extent of symptoms (clinical score) correlated strongly with the infection intensity (*R*^2^ = 0.80, S2 in Additional file 1). The recently developed two-tier disease severity threshold of 10 embedded fleas [17] aligned well with our data, clearly separating the majority of cases with less than 11 fleas and presenting with low clinical scores from those pupils with more than 10 fleas and higher clinical scores, hence severe disease.

The percent of infected pupils with severe disease in each county was correlated to the overall number of pupils infected in both surveys (*R*^2^ = 0.7, S3 in supplementary materials, Additional file 1) with the exception of Nakuru county (red dot in S3) where despite a relatively high number of cases (38) only 2.6% of the cases were severe (Table 3).

**Table 3:** Number of pupils examined, and percent infected for each affected school by county and survey

County	Survey 1			Survey 2		
	School ID	Number of pupils examined	Infection rate, %	School ID	Number of pupils examined	Infection rate, %
Turkana	159	107	0.9	45	110	1.8
	201	110	2.7	1086	109	0.9
	213	108	1.9			
	1101	110	1.8			
Samburu	101	108	0.9	130	111	5.4
	134	73	4.1	153	97	3.1
	141	111	2.7	169	105	1.0
	243	115	0.9			
	2164	128	10.2			
Kericho	157	116	1.7	143	113	1.8
	383	125	0.8	360	108	1.9
	461	113	2.7	433	104	1.0
	4014	119	1.7	533	108	0.9
Muranga	53	114	4.4	25	111	1.8
	193	113	1.8	106	112	0.9
	289	110	5.5	167	113	8.9
	327	112	2.7	300	111	5.4
	459	113	3.5	314	112	0.9
	472	112	3.6	392	113	0.9
	5121	111	0.9	432	114	0.9
				567	112	1.8
				5376	114	0.9
Nakuru	7	111	8.1	32	111	2.7
	212	112	0.9	62	109	3.7
	287	107	13.1	501	112	0.9
	390	111	6.3			
	6472	108	0.9			
Kajiado	3	109	2.8	39	109	0.92
	11	112	22.3	99	111	1.80
	77	128	2.3	257	114	0.88
	121	117	5.1	7116	105	1.90
Makueni	5	110	0.9	376	111	3.60
	329	114	0.9	814	110	0.91
	430	110	0.9			
	491	109	0.9			
	758	109	0.9			
	841	113	2.7			
Taita	177	110	0.9	107	112	0.89
				9116	108	0.93
Kilifi	14	110	0.9	110	110	7.27
	324	110	0.9	4007	109	9.17
	380	108	0.9	4316	107	0.93
	474	111	2.7	4353	109	0.92
	666	108	0.9	4370	108	3.70
				4088	110	5.45

### Evaluating rapid disease assessment

Observing only the tops of unwashed feet has been suggested as a rapid method for national surveys to assess tungiasis prevalence. However, of the 221 infected pupils for whom this was tested, only 82 (37.1%) had an infection that could be detected on the top of their feet. Using this rapid screening method would have missed 62.9% (*n* = 139) of all cases, 78.9% (*n* = 127) of 161 mild cases and 20.0% (*n* = 12) of 60 severe cases.

### Explanatory variables associated with tungiasis

Univariable analysis of data from the entire study population of examined pupils suggested that infection was associated with the county of residence, the school type, the class, age, sex and other skin conditions (Table 4). All of these variables were taken forward to the multivariable model except survey round and location type (urban/rural) as they were not associated with infection. The multivariable model highlighted the heterogeneity in disease prevalence between counties (Table 4) and demonstrated boys were twice as likely to be infected than girls. Children in public schools were five times as likely to be infected as those attending private schools (*aOR* = 5.62, 95% *CI*: 1.20–26.22). There was also a strong association between tungiasis infection and the presence of other skin conditions in examined pupils (*aOR* = 3.60, 95% *CI*: 2.38–5.45). There was no association of tungiasis infection with rural or urban location of the school (*OR* = 1.15, 95% *CI:* 0.49–2.70).

**Table 4:** Risk Factors for tungiasis in the whole pupil population (*n* = 21,246)

				Univariable				Multivariable final model			
Infection status		*n*	Actual infection rate, %	*OR*	95% *CI*		*P*	a*OR*	95% *CI*		*P*
Survey round	1	10,725	1.35	1							
	2	10,296	0.86	0.67	0.36	1.27	0.222				
County^1^	Muranga	2460	2.03	1				1			
	Turkana	2401	0.46	0.17	0.05	0.58	0.005	0.20	0.06	0.67	0.009
	Samburu	2349	1.32	0.38	0.12	1.19	0.096	0.34	0.11	1.04	0.059
	Kericho	2410	0.58	0.24	0.07	0.77	0.016	0.25	0.08	0.80	0.02
	Nakuru	2441	1.64	0.43	0.14	1.33	0.143	0.41	0.13	1.23	0.111
	Kajiado	2368	1.82	0.41	0.13	1.26	0.12	0.40	0.13	1.19	0.1
	Makueni	2439	0.53	0.20	0.06	0.68	0.009	0.19	0.06	0.60	0.005
	Taita Taveta	2412	0.12	0.04	0.01	0.22	< 0.001	0.05	0.01	0.26	< 0.001
	Kilifi	1967	1.58	0.57	0.18	1.79	0.337	0.66	0.22	1.99	0.463
Location type	Urban	3554	0.73	1							
	Rural	17,692	1.19	1.15	0.49	2.70	0.743				
School type	Private	1854	0.16	1				1			
	Public	19,392	1.20	6.51	1.31	32.21	0.022	5.62	1.20	26.22	0.028
Age				0.81	0.75	0.87	< 0.001	0.81	0.75	0.88	< 0.001
Sex	Girls	10,765	0.73	1.00				1			
	Boys	10,480	1.50	2.14	1.62	2.83	< 0.001	2.01	1.52	2.67	< 0.001
Disability	No	21,140	1.10	1.00							
	Yes	97	3.09	2.86	0.82	9.95	0.098				
Other skin disease	No	20,306	0.93	1.00				1			
	Yes	928	5.06	4.26	2.84	6.39	< 0.001	3.60	2.38	5.45	< 0.001

*OR* odds ratio, *CI* confidence interval, a*OR* adjusted odds ratio.^1^wald test for county *P* = 0.006

The estimated intra-cluster correlation coefficient for tungiasis was 0.40 (95% *CI*: 0.37–0.58) indicating a stronger correlation between individuals in a cluster than between clusters, which in this study were the schools.

Detailed interviews were held with a subsample of cases, including 330 boys (51%) and 313 girls (49%) with a mean age of 11.1 years (*SD* = 1.96). Only nine (1.4%) pupils in this group had a disability and 38 (5.9%) had another skin abnormality. Of the 643 interviewees, 77 (11.9%, 95% *CI*: 9.6–14.7%) were infected (S4 in Additional material, Additional file 1).

Univariable analyses identified several factors associated with tungiasis infection (Table 5). Variables that were significant predictors (*P* < 0.200) were taken forward to a multivariable model and retained in the multivariable model where *P* < 0.050 (Table 5). County and socio-economic status remained associated with tungiasis. Children who said they never use soap for foot washing had a nearly six times higher (*aOR* = 5.80, 95% *CI*: 1.08–31.17) odds of infection than those who say they always use soap. Pupils who say they wash their feet less than once a day had four times higher odds of infection (*aOR* = 4.64, 95% *CI*: 1.16–18.54) than those who said they wash more than once a day. Although not having a significant *P*-value, living in a house with floors of loose sand or soil was associated with infection (*aOR* = 2.57, 95% *CI*: 0.99–6.70) since dropping this variable from the model resulted in the AIC increasing (Table 5).

**Table 5:** Risk factors from the selected pupils in the second survey (*n* = 643)

		Univariable		Multivariable full model		Multivariable final model	
Tungiasis status	*n*	*OR* (95% *CI*)	*P*	a*OR* (95% *CI*)	*P*	a*OR* (95% *CI*)	*P*
County^1^							
Muranga	89	1		1		1	
Turkana	69	0.25 (0.05–1.30)	0.1	0.01 (0.00–0.10)	< 0.001	0.02 (0.00–0.12)	< 0.001
Samburu	67	0.41 (0.09–1.92)	0.256	0.04 (0.00–0.37)	0.005	0.03 (0.01–0.22)	< 0.001
Kericho	67	0.24 (0.04–1.27)	0.092	0.19 (0.03–1.25)	0.084	0.23 (0.04–1.30)	0.096
Nakuru	74	0.42 (0.09–1.93)	0.267	0.07 (0.01–0.48)	0.007	0.10 (0.02–0.61)	0.013
Kajiado	73	0.30 (0.06–1.47)	0.138	0.26 (0.04–1.78)	0.17	0.13 (0.02–0.76)	0.023
Makueni	72	0.41 (0.09–1.79)	0.236	0.15 (0.03–0.81)	0.028	0.15 (0.03–0.73)	0.019
Taita Taveta	67	0.06 (0.01–0.63)	0.02	0.06 (0.00–0.79)	0.033	0.05 (0,00–0.59)	0.018
Kilifi	65	2.19 (0.49–9.79)	0.305	0.05 (0.01–0.56)	0.014	0.12 (0.02–0.76)	0.024
Age		0.79 (0.68–0.91)	0.001	1.06 (0.88–1.27)	0.549		
Sex							
Girls	313	1		1			
Boys	330	1.95 (1.09–3.47)	0.024	1.35 (0.66–2.76)	0.414		
Disability							
No	634	1		1			
Yes	9	1.74 (0.24–12.47)	0.579				
Other skin disease							
No	605	1		1			
Yes	38	2.35 (0.84–6.59)	0.104	2.28 (0.61–8.48)	0.218		
Socioeconomic status		0.09 (0.03–0.25)	< 0.001	0.06 (0.03–0.35)	0.002	0.10 (0.03–0.33)	< 0.001
Shoes worn							
Closed W Socks	211	1		1			
None	75	6.24 (2.00–19.42)	0.002	3.10 (0.52–18.62)	0.217		
Slippers	152	2.74 (1.03–7.28)	0.043	2.00 (0.42–9.40)	0.378		
Closed	194	1.50 (0.65–3.46)	0.347	1.14 (0.35–3.67)	0.832		
Days attended school last week							
> 3 days	569	1		1			
1–3 days	69	2.54 (1.17–5.50)	0.018	2.09 (0.84–5.27)	0.12		
Soap use for feet washing^2^							
Always	378	1		1		1	
Never	20	8.60 (2.19–33.85)	0.002	5.23 (0.81–33.79)	0.082	5.80 (1.08–31.17)	0.041
Sometimes	243	2.46 (1.17–5.18)	0.018	1.25 (0.42–3.72)	0.685	1.66 (0.61–4.46)	0.319
Frequency of foot washing^3^							
More than once a day	333	1		1		1	
Once a day	265	2.07 (1.06–4.04)	0.034	2.15 (0.96–4.80)	0.062	2.40 (1.13–5.10)	0.023
Less than once a day	37	7.65 (2.30–25.44)	0.001	4.05 (0.91–17.93)	0.065	4.64 (1.16–18.54)	0.03
Bed net use							
Yes	275	1		1			
No	365	2.76 (1.40–5.43)	0.003	1.38 (0.59–3.21)	0.454		
Sleep in parent house							
No	128	1		1			
Yes	513	1.65 (0.73–3.70)	0.228				
Sleep on							
Bed	480	1		1			
Floor	160	2.28 (1.09–4.75)	0.028	1.03 (0.39–2.70)	0.956		
Floor material^4^							
Cement/Tiles	303	1		1		1	
Loose soil/sand	224	4.70 (2.13–10.39)	< 0.001	2.67 (0.92–7.72)	0.071	2.57 (0.99–6.70)	0.053
Hard mud/cow dung	116	1.85 (0.71–4.87)	0.211	1.03 (0.31–3.48)	0.957	0.82 (0.27–2.49)	0.723
Wall material							
Stone/cement	200	1		1			
Mud/natural	210	3.39 (1.43–8.03)	0.006	0.49 (0.12–2.02)	0.323		
Other	233	2.00 (0.82–4.88)	0.128	0.95 (0.27–3.30)	0.929		
Roof material							
Iron sheet	522	1		1			
Mud/natural	121	2.1 (0.89–4.99)	0.091	0.59 (0.17–2.14)	0.425		
Toilet type							
Improved	366	1		1			
Traditional latrine	178	3.86 (1.95–7.65)	< 0.001	1.41 (0.62–3.23)	0.414		
Open defecation	97	2.42 (0.92–6.4)	0.074	0.43 (0.10–1.83)	0.256		
Water source							
Own tap	194	1		1			
Open river/pond	225	2.15 (0.9–5.14)	0.086	0.59 (0.18–1.90)	0.374		
Shared tap/ well/borehole	223	1.51 (0.63–3.63)	0.355	0.74 (0.25–2.23)	0.596		
Parent attend school meetings							
Always	350	1		1			
Never	37	2.74 (0.81–9.32)	0.107	1.87 (0.35–9.86)	0.462		
Sometimes	253	1.5 (0.76–2.96)	0.238	1.25 (0.54–2.87)	0.604		
Parent ensure do homework							
Always	320	1					
Never	93	1.03 (0.42–2.56)	0.943				
Sometimes	229	1.71 (0.84–3.48)	0.141				
Family member ill some months							
No	539	1					
Yes	101	1.56 (0.69–3.50)	0.283				
Family owns a dog							
No	327	1.00		1			
Yes	317	0.29 (0.11–0.74)	0.009	0.55 (0.23–1.30)	0.172		
Family owns a cat							
No	346	1		1			
Yes	298	0.54 (0.25–1.16)	0.115	0.93 (0.43–2.01)	0.852		

*OR* odds ratio, *CI* confidence interval, a*OR* adjusted odds ratio. ^1^ wald test county* P* = 0.005 ^2^ wald test soap use *P* = 0.089 ^3^wald test frequency of foot washing *P* = 0.041 ^4^wald test floor material *P* = 0.030

## Discussion

Countries with endemic tungiasis struggle to design and target appropriate interventions due to the absence of data on country-wide disease burden, geographic distribution and characteristics of target communities. A number of small-scale studies that were specifically targeted in high burden settings [3, 10, 12] have shown that tungiasis can represent a major challenge for the primary health care system in Kenya. However, to establish data-informed guidelines and interventions, we needed to systematically assess the national disease burden of tungiasis in Kenya. To our knowledge, this is the first survey of this scale for any endemic country. We demonstrated that the national prevalence during the major rainy season in 2021 was 1.3%.

This is similar to another survey conducted in all households in the Kwale health and demographic surveillance system in 2011 [20], which found a prevalence of 1.1%. Other prevalence surveys that have been published to date report a wide range of prevalence from 7% in a village in coastal Kenya [12] to 63% in Napak district in northeastern Uganda [13]. These surveys did not randomly select clusters, but specifically targeted schools or villages previously known to have high numbers of cases. In addition, they were also mostly conducted during the dry season which has been associated with a higher prevalence in Brazil [10, 13, 21].

What is striking from the current survey is the high percentage (41%) of schools that have at least one case, indicating the disease is widespread across the country. The large difference in prevalence between counties and even between schools in the same county reflects the extreme heterogeneity in the distribution of tungiasis [3, 12, 22] which poses a considerable challenge for planning and targeting interventions.

Recently a new two-tier classification of mild versus severe disease was proposed [17], using the correlation of infection intensity with clinical signs. We applied this to the current population and confirmed a good fit of the proposed cut off point of 10 fleas as a suitable threshold to separate cases with low and high levels of morbidity. This classification revealed the proportion of cases with severe disease correlates with the number of cases in a county, and by inference, prevalence of disease. The unusually low proportion of cases with severe disease in Nakuru could be the result of past interventions in the county (County Health Management Team personal communication). When treatment is always available in a community, people can obtain treatment as soon as they are newly reinfected and therefore will not accrue a high intensity of infection, as seen during a community-based effort to control tungiasis in Kenya [23].

We assessed SES from our interview data and demonstrated a very strong association. Living in poverty exposes people to other risk factors such as living in a house with an unsealed earthen floor, and/or lacking access to water and soap. The association with attending a public school rather than a private one is probably also related to SES, but in addition, private schools may have better access to water and the teachers and parents pay more attention to the personal hygiene of pupils. All possible explanations that require further investigation. Likewise, the association with other skin abnormalities is likely to be linked to SES, for example, through shared risk for scabies, head lice and/or fungal infections [24].

The possible protective role and mechanism of at least daily foot washing with soap needs further investigation, as does the higher odds of infection for boys, which is a common finding in all tungiasis surveys. Risk in boys could be due to behaviours that put them more at risk of infection, differing behaviour of caregivers, or as yet unknown biological factors.

Anecdotal reports suggest people with disabilities, either physical or mental, living in endemic areas have a high infection intensity. In the current study we attempted to identify pupils with disabilities without the use of a formal tool. Our data seem to support this, but the small sample size resulted in a wide confidence interval, hence further research should specifically investigate the risk associated with disability.

One limitation of this survey was the low number of infected cases available to enrol for the risk factor interviews that resulted in the large confidence intervals. A second limitation was the protracted time it took for all counties to complete the second survey which meant we could not obtain a second prevalence estimate in a different season. The delay in some counties was caused by delays in mobilising funds for the team which were exacerbated by campaigns and national elections in 2022. Another limitation could have been the use of school-based surveys which could miss children from the poorest families who cannot afford the costs to attend school, or those with such severe disease they were unable to walk to school due to the pain. Nevertheless, our surveys did include children with low SES as assessed here (10% of the interviewed pupils) and 26% of infected pupils had severe disease. Consequently, we suggest a public school-based strategy is likely to be a scalable and cost-effective means for mapping infected communities.

The process of washing, drying and thorough examination of feet is time consuming, but we have demonstrated that replacing it with a quick look at the top of feet alone, as suggested previously [14] would miss two thirds of infected children in this population. Instead, we would recommend further research to test a method that would quickly look at both the bottom and top of the feet, only washing those that are very dirty. This could be conducted by trained teachers or community health workers linked to the school.

## Conclusions

Findings from this survey confirm our previous observations that tungiasis could be reduced through a one health approach addressing treatment, control of off-host stages in unsealed earthen house floors and improved personal hygiene. Animal interventions may be required in some communities, but neither the current study nor previous ones in Kenya have demonstrated a role for animals in transmission in Kenya. Currently large-scale WASH (water, sanitation and hygiene) programs are conducted globally focussing entirely on handwashing, but if this messaging could change to whole body washing with soap, they could address multiple diseases, including tungiasis. Interventions retrofitting low-cost floors into houses of infected families need to be explored and their efficacy compared with WASH interventions which may be more affordable but have a short-term impact. The link with poverty is typical for many infectious diseases and re-emphasizes the need for integrated and community-based development and disease control programs.

### Supplementary Information

**Additional file 1: S1:** Flow diagram describing selection of participant groups. Orange boxes represent pupils infected with tungiasis; green boxes represent uninfected pupils. Blue arrows depict flow in the first survey, orange arrows depict flow in the second survey. **S2** Correlation of infection intensity with clinical score for all pupils with tungiasis in their feet (*n* = 242) with a fitted linear regression line (dotted blue line). The red line indicates the 10-flea threshold for mild and severe disease. **S3** Correlation of county percent infected with percent of cases with severe disease (*n* = 225)**. S4** Demographics of pupils selected for risk factor interviews**. S5** Polychoric Principal Component Analysis for a Socio-Economic Variable*,* Cronbach alpha and correlation with county poverty rate.

## Data Availability

The datasets and other materials supporting the conclusions of this article are available on KWTRP Research Data Repository at Harvard Dataverse through the following link: https://doi.org/10.7910/DVN/DFSTIZ.

## Abbreviations

a*OR*: Adjusted odds ratio
CHMT: County Health Management Team
*CI*: Confidence interval
KEMRI: Kenya Medical Research Institute
NTDs: Neglected tropical diseases
*OR*: Odds ratio
PTA: Parent Teachers Association
SES: Socio-economic status
*SD*: Standard deviation
WASH: Water, sanitation and hygiene
