# Supplementary material for: National prevalence and risk factors for tungiasis in Kenya
Source: Infect Dis Poverty. 2023 Sep 18;12:85. doi: 10.1186/s40249-023-01131-x (PMC10506256; doi:10.1186/s40249-023-01131-x)
Supplement: Supplementary file 1 — Additional file 1: S1: Flow diagram describing selection of participant groups. Orange boxes represent pupils infected with tungiasis; green boxes represent uninfected pupils. Blue arrows depict flow in the first survey, orange arrows depict flow in the second survey. S2 Correlation of infection intensity with clinical score for all pupils with tungiasis in their feet (n = 242) with a fitted linear regression line (dotted blue line). The red line indicates the 10-flea threshold for mild and severe disease. S3 Correlation of county percent infected with percent of cases with severe disease (n = 225). S4 Demographics of pupils selected for risk factor interviews. S5 Polychoric Principal Component Analysis for a Socio-Economic Variable, Cronbach alpha and correlation with county poverty rate. [file 40249_2023_1131_MOESM1_ESM.docx]

# National prevalence and risk factors for tungiasis in Kenya

## Additional file 1: Materials

9 counties

First survey

randomly selected

99 schools

Second survey

randomly selected

97 schools

School pupils aged 8–14 years

Randomly selected, *n* = 10,865

School pupils aged 8–14 years

Randomly selected, *n* = 10,600

Infected

*n* = 147

Uninfected

*n* = 10,718

Infected

*n* = 95

Uninfected

*n* = 10,505

Infected

*n* = 77

Selected for risk factor interview

Uninfected

*n* = 566

Infected

*n* = 242

Population-wide risk factor analysis

Uninfected

*n* = 21,223

Infection intensity & morbidity assessment *n* = 242

Infected

*n* = 82

Rapid assessment *n* = 221

Uninfected

*n* =139

**S1** Flow diagram describing selection of participant groups. Orange boxes represent pupils infected with tungiasis; green boxes represent uninfected pupils. Blue arrows depict flow in the first survey, Orange arrows depict flow in the second survey.


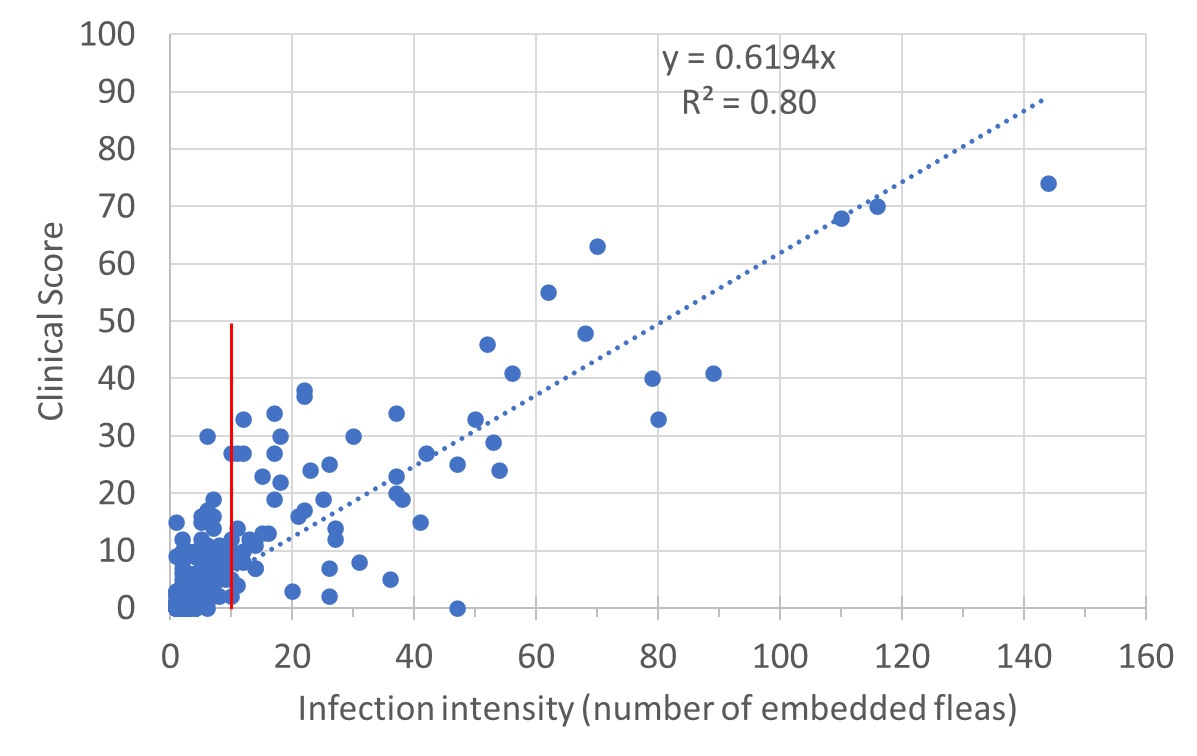


**S2** Correlation of infection intensity with clinical score for all pupils with tungiasis in their feet (n = 242) with a fitted linear regression line (dotted blue line). The red line indicates the 10 flea threshold for mild and severe disease.


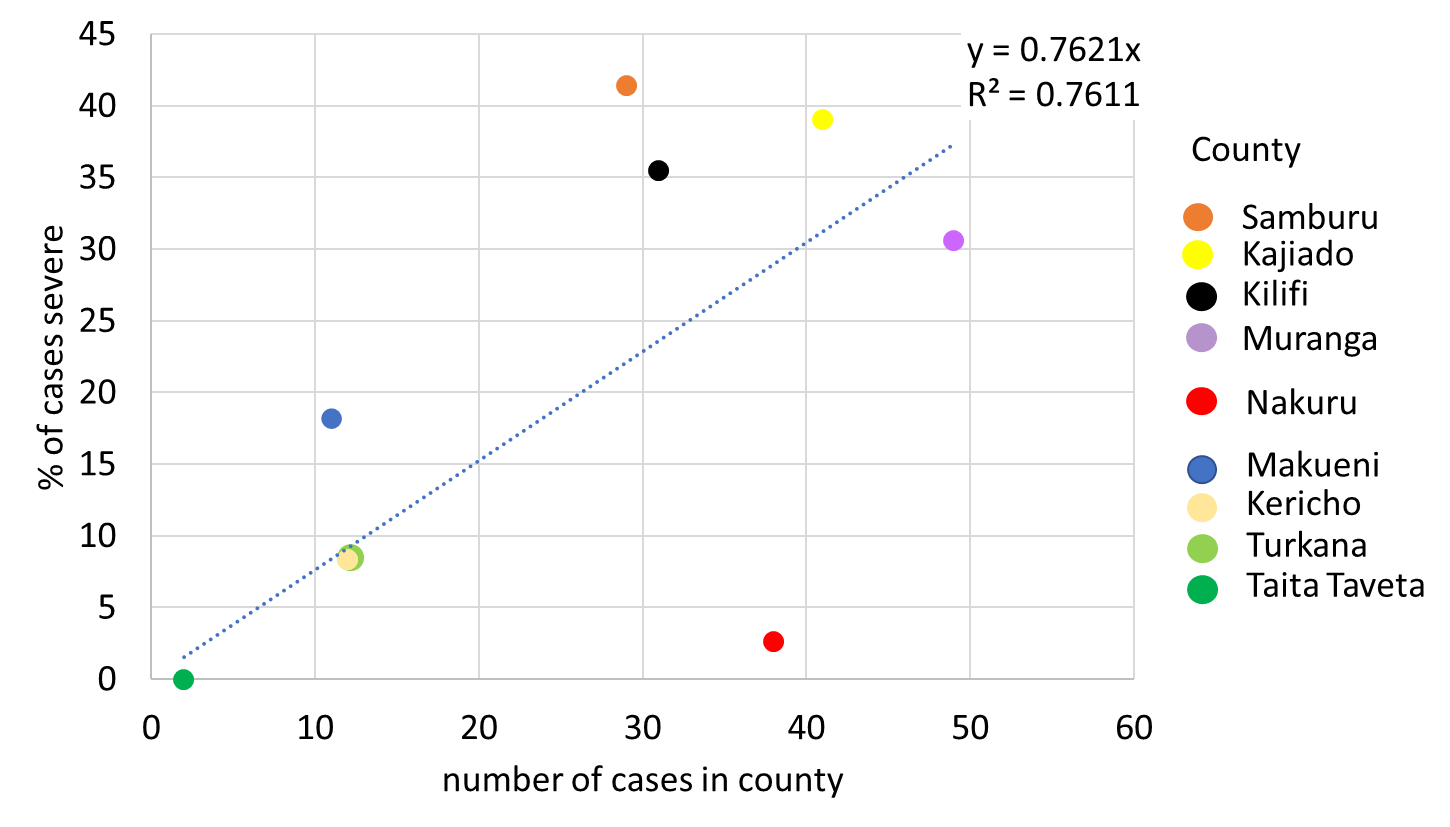


**S3** Correlation of county percent infected with percent of cases with severe disease (n = 225)

**S4** Demographics of pupils selected for risk factor interviews

| Months of survey | Oct 2021–Oct 2022 |
| --- | --- |
| Number of schools | 95 |
| Number of pupils selected | 643 |
| % boys | 51.3 |
| Mean age | 11.1 |
| Number with disability (%)^a^ | 9 (1.4) |
| Number with other skin abnormality (%) ^a^ | 38 (5.9) |
| Number with tungiasis (%) | 77 (11.9) |

^a^ not assessed by a clinician

**S5** Polychoric Principal Component Analysis for a Socio-Economic Variable

To construct the socio-economic status (SES) variable, polychoric principal component analysis was conducted with variables on ownership of household and individual assets/items such as wall materials, family income source and water source (Table S4 in supplementary materials. This was conducted by adding and removing potential variables ensuring all had an item-rest correlation in Cronbach’s alpha > 0.1 to obtain a factor with an overall Cronbach’s alpha >0.7 (See S5 in supplementary materials). The floor materials variable was excluded from the SES variable as it could be biologically associated to the dependent variable (disease status) in risk factor analysis. To confirm the SES factor correlated with national indicators a scatterplot and regression were conducted of county mean SES versus the county % of population living under the national poverty level which had an *R*^2^ of 0.576 ([8], S6 supplementary material).

Variables included in the final Socio-Economic Factor generated through Principal Component Analysis

|  | Variable |
| --- | --- |
|  | Pupil wearing a uniform (none/partial/full) |
|  | Condition of the clothes (poor/medium/ good) |
|  | Type of shoes (none/open sandals/ closed/closed with socks) |
|  | Family income from a job (yes/no) |
|  | House wall materials (mud with sticks and or stones, palm leaves, grass, wood/iron sheets/compressed mud bricks/stone bricks with cement) |
|  | Water source (open/ shared tap/own tap) |
|  | Family owns tv (yes/no) |
|  | Family owns mobile phone (yes/no) |
|  | Family owns sofa (yes/no) |
|  | Family owns cupboard (yes/no) |
|  | Own no animals (yes/no) |

Cronbach’s alpha for Socio-Economic Factor

| Item | Obs | Sign | Item-test correlation | Item-rest correlation | Interitem correlation | Alpha |
| --- | --- | --- | --- | --- | --- | --- |
|  |  |  |  |  |  |  |
| Wearing uniform | 637 | + | 0.6030 | 0.4820 | 0.2436 | 0.7631 |
| Condition of clothes | 616 | + | 0.5920 | 0.4685 | 0.2453 | 0.7647 |
| Type of shoes | 632 | + | 0.7010 | 0.6028 | 0.2305 | 0.7497 |
| Family income from job | 643 | + | 0.4801 | 0.3402 | 0.2609 | 0.7793 |
| Wall materials | 642 | + | 0.6412 | 0.5297 | 0.2385 | 0.7580 |
| Water source | 642 | + | 0.4911 | 0.3536 | 0.2590 | 0.7776 |
| Family own tv | 643 | + | 0.7084 | 0.6123 | 0.2292 | 0.7484 |
| Family own mobile phone | 643 | + | 0.4277 | 0.2812 | 0.2682 | 0.7856 |
| Family own sofa | 643 | + | 0.6811 | 0.5785 | 0.2331 | 0.7524 |
| Family own cupboard | 643 | + | 0.6335 | 0.5206 | 0.2397 | 0.7592 |
|  | 643 | + | 0.2456 | 0.0853 | 0.2930 | 0.8056 |
| Test scale |  |  |  |  | 0.2492 | 0.7850 |


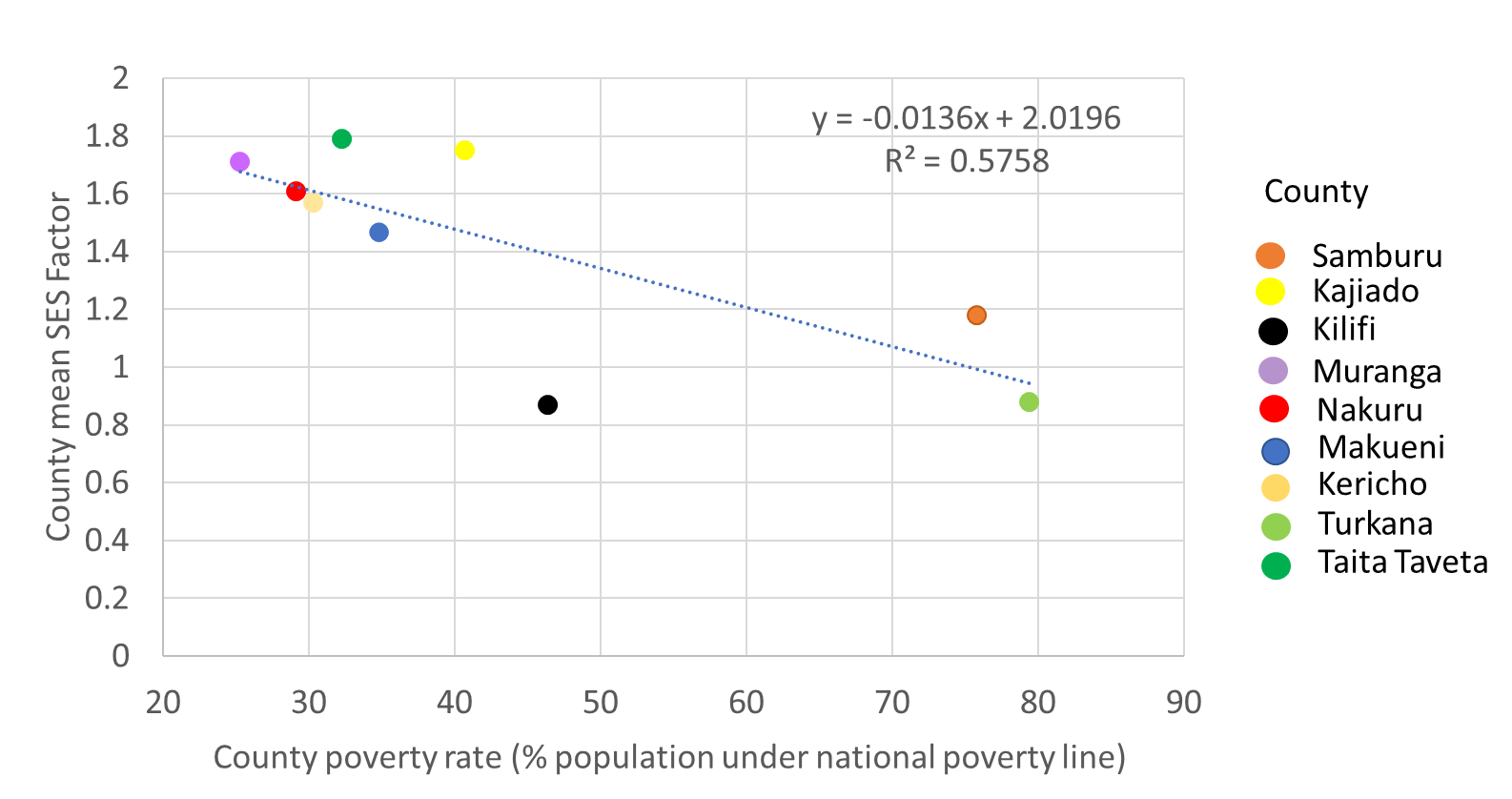


Correlation of SES factor with county poverty rate
